# Improved species assignments across the entire *Anopheles* genus using targeted sequencing

**DOI:** 10.3389/fgene.2024.1456644

**Published:** 2024-09-19

**Authors:** Marilou Boddé, Alex Makunin, Fiona Teltscher, Jewelna Akorli, Nana Efua Andoh, Amy Bei, Victor Chaumeau, Ife Desamours, Uwem F. Ekpo, Nicodem J. Govella, Jonathan Kayondo, Kevin Kobylinski, Elhadji Malick Ngom, El Hadji Amadou Niang, Fredros Okumu, Olaitan O. Omitola, Alongkot Ponlawat, Malala Nirina Rakotomanga, Mialy Tatamo Rasolonjatovoniaina, Diego Ayala, Mara Lawniczak

**Affiliations:** ^1^ LIB Leibniz Institute for the Analysis of Biodiversity Change, Center for Molecular Biodiversity Research, Bonn, Germany; ^2^ Institut Pasteur de Madagascar, Medical Entomology Unit, Antananarivo, Madagascar; ^3^ Wellcome Sanger Institute, Tree of Life, Hinxton, United Kingdom; ^4^ Department of Parasitology, Noguchi Memorial Institute for Medical Research, Accra, Ghana; ^5^ Department of Pathology, University of Cambridge, Cambridge, United Kingdom; ^6^ School of Public Health, Yale University, New Haven, CT, United States; ^7^ Shoklo Malaria Research Unit, Mahidol Oxford Tropical Medicine Research Unit, Faculty of Tropical Research, Mahidol University, Mae Ramat, Thailand; ^8^ Nuffield Department of Medicine, Centre for Tropical Medicine and Global Health, University of Oxford, Oxford, United Kingdom; ^9^ Department of Pure and Applied Zoology, Federal University of Agriculture, Abeokuta, Nigeria; ^10^ Ifakara Health Institute, Ifakara, Tanzania; ^11^ Uganda Virus Research Institute, Entebbe, Uganda; ^12^ Armed Forces Research Institute of Medical Science, Bangkok, Thailand; ^13^ Institut Pasteur de Dakar, Dakar, Senegal; ^14^ Laboratory of Vector and Parasite Ecology, Cheikh Anta Diop University, Dakar, Senegal; ^15^ Infectious Diseases and Vectors: Ecology, Genetics, Evolution and Control (MIVEGEC), University Montpellier, National Centre for Scientific Research (CNRS), Research and Development Institute (IRD), Montpellier, France

**Keywords:** vector monitoring, malaria vector, species assignment, *Anopheles*, amplicon panel

## Abstract

Accurate species identification of the mosquitoes in the genus *Anopheles* is of crucial importance to implement malaria control measures and monitor their effectiveness. We use a previously developed amplicon panel (ANOSPP) that retrieves sequence data from multiple short nuclear loci for any species in the genus. Species assignment is based on comparison of samples to a reference index using *k*-mer distance. Here, we provide a protocol to generate version controlled updates of the reference index and present its latest release, NNv2, which contains 91 species, compared to 56 species represented in its predecessor NNv1. With the updated reference index, we are able to assign samples to species level that previously could not be assigned. We discuss what happens if a species is not represented in the reference index and how this can be addressed in a future update. To demonstrate the increased power of NNv2, we showcase the assignments of 1789 wild-caught mosquitoes from Madagascar and demonstrate that we can detect within species population structure from the amplicon sequencing data.

## Introduction

The genus *Anopheles* contains many species complexes: groups of closely related species which share large amounts of genetic variation and can sometimes hybridise in areas of sympatry (Harbach and Kitching, 2016; Anopheles gambiae 1000 Genomes Consortium et al., 2017). Usually there are no known morphological keys to distinguish between species inside species complexes and molecular methods are required. Most commonly used are species diagnostic PCRs, which target a single locus and determine the species based on the length of the amplified sequence (Scott et al., 1993; Fanello et al., 2002; Cohuet et al., 2003; Wilkins et al., 2006). The target sites and primers used in these PCRs are specific to each species complex and failures (lack of a band) are difficult to interpret (Erlank et al., 2018).

Within the genus *Anopheles*, which contains approximately 500 described species, a few dozen species are known vectors of human malaria (Wilkerson et al., 2021). Apart from vector capacity, the species within this genus also greatly vary in their range, host preference, biting behaviour and insecticide resistance profiles (Carnevale and Robert, 2009). Detailed knowledge of each species’ contribution to malaria transmission and how this changes through space and time is crucial to design and assess effective malaria control measures (Krzywinski and Besansky, 2003; Erlank et al., 2018). Currently, *Anopheles* species identification heavily relies on morphological assessments, which are time consuming and where the accuracy depends on the level of expertise of the taxonomist. Morphological identification keys are often specific to a certain geographic region (Le Goff et al., 2012; Gunathilaka, 2017; Coetzee, 2020; Sallum et al., 2020) and rare species or those invading from other regions, a phenomenon that might become more common due to climate change, might be misidentified and go undetected for some time.

ANOSPP (ANOpheles Species and Plasmodium Panel) is an amplicon sequencing approach that targets up to 62 nuclear loci in the generic *Anopheles* genome to determine the species together with two mitochondrial loci in the generic *Plasmodium* genome to assess parasite presence in mosquitoes. It was built to address the challenges mentioned above and can be used for robust and accurate species identification for mosquitoes collected globally (Makunin et al., 2022). ANOSPP can be used for large scale monitoring of Anopheles to understand species diversity and potential transmission patterns and in comparison to whole genome sequencing, it operates at a small fraction of the cost. Indeed, ANOSPP can also be used as a first step analysis to identify specimens of interest for whole genome data generation.

To assign mosquito species, the obtained sequence data is compared to a reference index using *k*-mer distances (Boddé et al., 2022). For each of the sample’s target haplotypes, we identify its nearest neighbour in the reference index as the haplotype that minimises the *k*-mer distance. We normalise over alleles and targets to obtain the sample’s assignment proportions over all the species represented in the reference index, based on the species labels of the nearest neighbours.

The reference index contains sequence data of up to ten individuals per species, where we carefully assessed the accuracy of the species label. We are in the process of sequencing many thousands of mosquitoes with the ANOSPP panel. Among those, we encountered species that were not represented in our initial reference index, NNv1, and as a result some mosquitoes could not be assigned to the correct species. We aim to regularly update the ANOSPP version controlled reference index to enable more accurate discernment of species identity. Here we discuss the process of updating the reference index together with the latest release, NNv2.

The genus *Anopheles* is divided into seven subgenera, five of which contain relatively few species, while the subgenera *Anopheles* and *Cellia* contain more than 200 species each (Harbach, 2013; Harbach and Kitching, 2016). These two subgenera, as well as the subgenus *Nyssorhynchus* that contains over 30 described species, are divided into sections and series, which contain further divisions into groups, subgroups and complexes. Although the taxonomic units between subgenus and species are not officially recognised as scientific classification levels (International Commission on Zoological Nomenclature, 1999; Harbach, 2013), historically they have been widely used in the *Anopheles* taxonomy. The taxonomic classification is based on morphological traits and does not always agree with phylogenetic relationships between species, inferred from DNA markers (Harbach, 2013).

Mirroring the hierarchical classification of the genus *Anopheles*, the samples in the reference index are grouped at three different levels. Roughly, the fine level in the reference index corresponds to species in the taxonomy; the intermediate level corresponds to groups, subgroups or complexes in the taxonomy; and the coarse level corresponds to series for the subgenera *Anopheles* and *Cellia* and to subgenus for the other subgenera. At the fine level, NNv2 contains 91 of approximately 500 described *Anopheles* species (Wilkerson, Linton and Strickman, 2021). At the intermediate and coarse level respectively, 53 groups, subgroups and complexes and 11 series and subgenera are represented.

We arrived at NNv2 through an iterative process of adding and removing samples and revising species labels. Where available, we took the species labels provided by our sample partners, mostly based on morphological identifications, as a starting point. However, the accuracy of morphological identification depends on the level of expertise of the taxonomist and can be undermined by cryptic genetic variation, hybridisation and shifting species ranges. Moreover, morphological identifications often lack the resolution to distinguish between species within a species complex or group. Therefore, it is necessary to validate these species labels by other means. We assessed the confidence of the species labels by comparing them with clustering results on ANOSPP sequence data, by assessing the taxonomic placement of a species cluster, and by considering sequence similarity and taxonomic placement of ITS2 barcode sequences generated from relevant samples. Here we also introduce a naming convention to highlight any conflicts between these lines of evidence. To showcase the power of ANOSPP and the improvements offered through the NNv2 reference index, we provide an exploration of 1789 samples from Madagascar and demonstrate that ANOSPP is able to capture geographic signal within certain species.

ANOSPP panel development (Makunin et al., 2022) and the analytical approach (Boddé et al., 2022) have now provided the framework to support the expansion of species diversity exploration in this medically important genus of human malaria vectors. Here we show that increasing the number of species represented in the reference index greatly improves species assignment and regular updates of the reference index are crucial to accurately monitor vector populations.

## Materials and methods

### Sample processing

A total of 2163 mosquitoes were collected from several locations using a variety of collection methods; see Supplementary Tables S1–S3 for information for each individual mosquito. Samples were shipped to Sanger, where they were processed and sequenced according to the protocol in Makunin et al. (2022); Korlević et al. (2021); Makunin et al. (2022). Sequence data were processed as in Boddé et al. (2022); Makunin et al. (2022). Species assignment was performed using NNoVAE (Boddé et al., 2022) using standard parameters with the following exceptions. The assignment threshold was changed from 0.8 to 0.7 to obtain a higher assignment rate for closely related species; e.g., individuals assigned to Funestus_subgroup_cl1_f§ tend to have assignment proportions between 0.7 and 0.8 for Funestus_subgroup_cl1_f§. Assignments are still robust, because with a 0.7 threshold the difference in assignment proportion between the highest and second highest proportion is at least twofold. We also introduced new contamination risk categories, including ‘low’ (≥1,000 reads and 0 multiallelic targets), ‘medium’ (<1,000 reads or one to two multiallelic targets), ‘high’ (3 or four multiallelic targets) and ‘very high’ (more than four multiallelic targets).

#### ITS2 sequencing

ITS2 barcodes were sequenced for a few individuals for each species (Supp 4); ideally after species assignment, but sometimes we used the morphological labels to select samples before ANOSPP sequencing. ITS2 sequences were generated as in Makunin et al. (2022), using ITS2A (5′-TGT​GAA​CTG​CAG​GAC​ACA​T-3′) and ITS2B (5′-TAT​GCT​TAA​ATT​CAG​GGG​GT-3′) primers for PCR amplification (Beebe and Saul, 1995) and sent for Sanger sequencing using the ITS2A primer and the Eurofins GATC SupremeRun 96 service (https://eurofinsgenomics.eu/en/custom-dna-sequencing/gatc-services/supremerun-plate/). We used cutadapt 4.6 (Martin, 2011) to remove incomplete ITS2 sequences by requiring the ITS2B primer to be present at the end of the sequence. Selected samples with incomplete ITS2 sequences were resequenced from the ITS2B primer; for those samples consensus sequences were generated using the DNAsubway (Hilgert et al., 2014). Sequence completeness was assessed by looking at multiple sequence alignments within a group of related species and incomplete sequences were removed manually.

#### Tree generation

We generated trees from ITS2 sequences using selected samples from multiple species and ANOSPP sequence trees for the samples in NNv2. Multiple sequence alignment was performed on ITS2 sequences or on ANOSPP sequences for each target separately using mafft v7.520 (Katoh and Standley, 2013). Trees were generated with FastTree version 2.1.11 using the -nt option (Price et al., 2009). The trees from different ANOSPP targets were combined into a species tree using astral version 5.7.8 (Zhang et al., 2018). Tree visualisation was done in iTOL (Letunic and Bork, 2024).

### Reference index creation

The candidate samples for NNv2 included the samples from NNv1 (Boddé et al., 2022), additional individuals of the species already represented in NNv1 and individuals with species labels that were not present in NNv1 (Small et al., 2020). We selected up to 10 individuals per species, collected from as many different countries as possible, but not more than five individuals from the same country. For species with a large geographic distribution that is well represented in the datasets we have analysed to date, that means NNv2 includes only one or two individuals per country (e.g., for An. arabiensis we included one or two individuals from seven countries, such that the total number of individuals equals 10), whilst for species with a narrow geographic range or where we have processed samples from a limited number of locations, NNv2 includes only five individuals, all from the same country (e.g., Five An. pauliani from Madagascar, 1 An. obscurus from Gabon). In this way we try to capture within species variation, while keeping the reference index lightweight and assignments computationally feasible. We used the assignment results obtained using NNv1 to avoid clearly misidentified individuals, for example, of the 44 individuals morphologically identified as An. mascarensis, three were assigned to Anopheles_maculipalpis using NNv1. An. maculipalpis and An. mascarensis are quite distantly related (Figure 2), so we concluded that those three individuals were morphologically misidentified.

For the selected set of individuals we computed the *k*-mer distance between each pair of samples from the ANOSPP target sequences [see Boddé et al. (2022) for the precise mathematical definition]. These pairwise distances are used to identify clusters of samples, referred to as species-groups, by setting thresholds on the normalised *k*-mer distance. As in Boddé et al. (2022), we used a threshold of 0.1 for the fine level species-groups and 0.3 for the intermediate level species-groups. Although these thresholds do not partition the set of candidate samples perfectly (see Supplementary Figures S1–S7), the clustering agrees reasonably well with the species and complex, subgroup or group labels respectively and is sufficient as a starting point to define species-groups. It was previously shown that there is not a single threshold that clearly separates all series and subgenera (Boddé et al., 2022). Therefore we decided to define the coarse level species-groups in accordance with the taxonomic series for the species-rich subgenera, *Anopheles* and *Cellia*, and with the taxonomic subgenus for the subgenera *Nyssorhynchus* and *Kerteszia*, rather than based on a threshold.

Some closely related species (e.g., *An. coluzzii* and *An. gambiae*; and *An. epiroticus* and *An. sundaicus*) have *k*-mer distances below the fine level threshold; an indication that the NN method on all ANOSPP targets cannot confidently distinguish between them. In this case a species-group represents multiple species (there are seven such species-groups with labels ending in §; see Table 1) and in order to distinguish between them a follow up method has to be implemented [e.g., the variational autoencoder (VAE) for the Gambiae Complex, see Boddé et al. (2022)]. On the other hand, sometimes we see several clusters at the fine level threshold of samples with the same species label. This likely represents an undescribed species complex (e.g., *An. pharoensis*) or considerable geographic structure (e.g., *An. squamosus*). In some cases we include the clusters as separate species-groups, indicated by the species name followed by _cl1, _cl2, etc., (as for *An. pharoensis*), where “cl” stands for clade. In other cases we merge the clusters into a single species group (as for *An. squamosus*). The decision as to whether a species is represented by multiple clusters depends on the distance between members of the clusters, whether most individuals of the species can be clearly assigned to one cluster or whether many individuals have mixed assignments, whether individuals from different clusters occur sympatrically and whether ITS2 information supports the clustering.

**TABLE 1:** Species-groups with uncertainty or potential conflict.

Level	Species-group	Issue
Intermediate	An_rhodesiensis_i*	NN coarse: Myzomyia series Taxonomy: Neomyzomyia series
	Marshallii_group_i*	Contains An_theileri* (taxonomy: Wellcomei group) and An_mascarensis* (taxonomy: Mascarensis group, Neomyzomyia series)
	Moucheti_complex_i*	Contains An_jebudensis* (taxonomy: Smithii group, Neomyzomyia series)
	Sundaicus_subpictus_ complex_i§	Taxonomy: Sundaicus and Subpictus are separate complexes in the Pyretophorus series
	Myzomyia_series_cl1_i^	Unnamed species in Myzomyia series
Fine	An_rhodesiensis*†	NN coarse: Myzomyia series Taxonomy: Neomyzomyia series Also strong geographic structure
	An_theileri*	NN intermediate: Marshallii group Taxonomy: Wellcomei group
	An_mascarensis*	NN intermediate, coarse: Marshallii group, Myzomyia series Taxonomy: Mascarensis group, Neomyzomyia series
	An_jebudensis*	NN intermediate, coarse: Moucheti complex, Myzomyia series Taxonomy: Smitthii group, Neomyzomyia series
	Funestus_subgroup_cl1_f§	Contains *An. funestus, An. funestus-like* and *An. vaneedeni*
	Funestus_subgroup_cl2_f§	Contains *An. longipalpis* and *An. parensis*
	Marshallii_group_cl1_f§	Potentially contains *An. marshallii*, *An. hancocki* and *An. brohieri*
	Marshallii_group_cl2_f§	Potentially contains *An. marshallii*, *An. hancocki* and *An. brohieri*
	Marshallii_group_cl3_f§	Potentially contains *An. marshallii*, *An. hancocki* and *An. brohieri*
	An_gambiae_coluzzii§	Contains *An. gambiae* and *An. coluzzii*
	Sundaicus_complex_f§	Contains *An. sundaicus* and *An. epiroticus*
	Coustani_group_cl1_f§	Potentially contains *An. coustani* and *An. tenebrosus*
	Coustani_group_cl2_f§	Potentially contains *An. coustani* and *An. tenebrosus*
	Myzomyia_series_cl1_f^	Unnamed species in Myzomyia series
	Rivulorum_subgroup_cl1_f^	Unnamed species in Rivulorum subgroup
	Christya_series_cl1_f^	Unnamed species in Christya series
	Leucosphyrus_group_cl1_f^	Unnamed species in Leucosphyrus group
	An_squamosus†	Strong geographic structure

#### Naming convention

The fine (0.1 *k*-mer distance threshold) and intermediate (0.3 *k*-mer distance threshold) level species-groups are named according to the species they represent, suffixed by _cl1, etc. if necessary. If a species-group (meaning a cluster of samples that are grouped based on these thresholds) represents several species, the species-group is named according to the species complex, subgroup or group to which these species belong, again suffixed by _cl1, etc. if necessary. If a species-group represents one or more unnamed species, it is named according to the complex, subgroup or group it most likely belongs to as deduced from the pairwise distances on the ANOSPP data, always followed by _cl1, etc. Fine level and intermediate level species groups are suffixed by _f and _i respectively, with the exception of fine level species-groups with a one-to-one correspondence to a single species, in which case the _f suffix is omitted (this is the case for 65 of 91 fine level species-groups). Coarse level species-groups are named according to the taxonomic series they represent for the subgenera *Anopheles* and *Cellia* and according to the subgenus for the subgenera *Kerteszia* and *Nyssorhynchus*. If there is a conflict between ANOSPP data and taxonomic placement, the species-group name is appended with an *. If a fine level species-group consists of several named species or an intermediate level species-group consists of several named complexes, the species-group name is appended with an §. Species-groups representing unnamed species are appended by a ^. Species-groups that show evidence of strong geographical structure are appended by †. Table 1 provides detail on each affected species-group with these symbols appended. Species-group labels without any suffixes are highly confident.

## Results

### Identified species-groups

**FIGURE 1:**
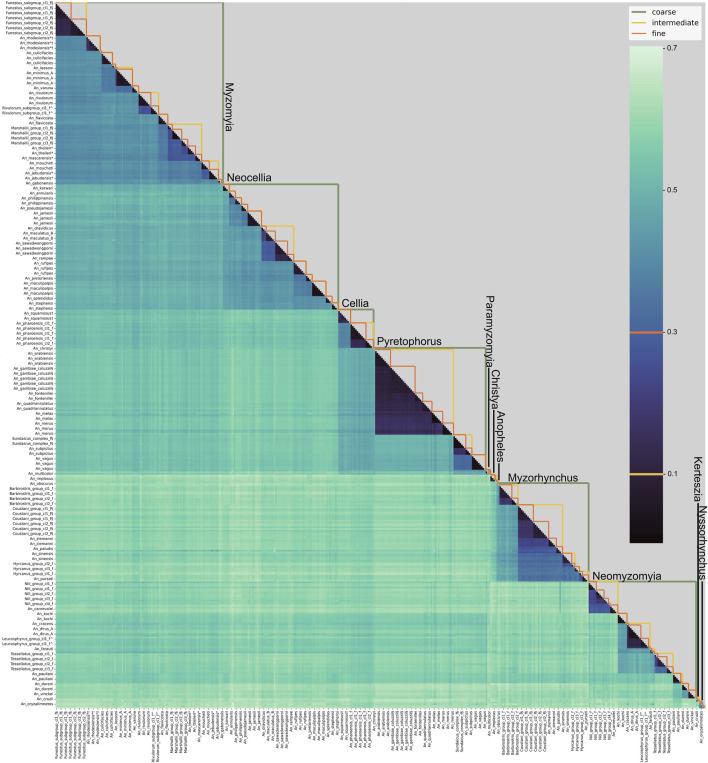
Pairwise distances between samples in NNv2. The heatmap shows the pairwise *k*-mer distance between all individuals in NNv2. Individuals are in the same order on the horizontal and vertical axes and are labelled by the fine-level species groups (labels are shown only for every third sample because of size restrictions). The orange (threshold 0.1), yellow (threshold 0.3) and green lines in the upper triangle indicate the fine, intermediate and coarse level species groups respectively. The coarse level species-group labels are displayed on the figure.

**FIGURE 2:**
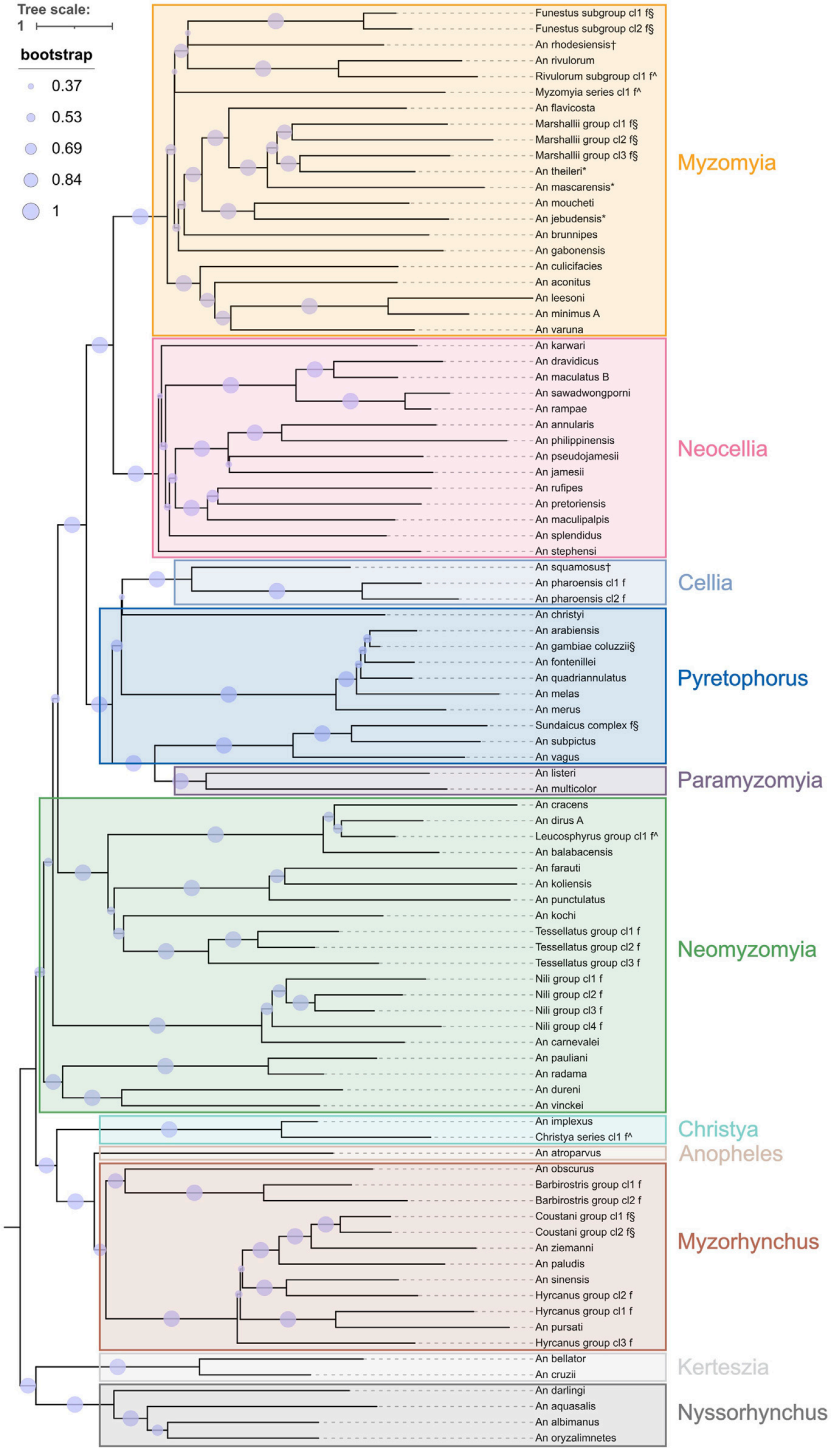
Species tree from ANOSPP target sequence. Separately generated trees for each target were merged into a single species tree using ASTRAL. The leaves are labelled by the fine level species-groups and the coarse level species-groups are indicated with coloured boxes. Branches are scaled by substitution rate and bootstrap support values are indicated by the size of the purple circles.

The eleven coarse level species-groups follow the taxonomic series within the subgenera *Anopheles* and *Cellia* and represent the entire subgenus for the subgenera *Nyssorhynchus* and *Kerteszia*. The normalised pairwise *k*-mer distances largely support this division (Figure 1; Supplementary Figure S1). It is worth noting that the Cellia Series does not form a separate cluster from the Pyretophorus Series and exploration of the phylogenetic relationships of *An. squamosus* and *An. pharoensis* with the species in the Pyretophorus Series should be undertaken. Pairwise *k*-mer distances also reveal that the Neomyzomyia Series consists of at least four separate clusters; consistent with several phylogenetic studies which reported that the Neomyzomyia Series is non-monophyletic (Foley et al., 1998; Harbach, 2013; Makunin et al., 2022). The species tree based on alignments of ANOSPP sequence data also indicates that the Pyretophorus Series is paraphyletic with respect to the Cellia and Paramyzomyia Series (Figure 2). The species tree also supports the finding that the Neomyzomyia Series is non-monophyletic.

The 53 species-groups at the intermediate level (0.3 threshold) typically represent groups, subgroups or complexes within the genus. However, if the intermediate level species-group in the reference index contains a single fine level species-group, it is labelled with the species name and not by the taxonomic unit that species represents. Intermediate level species groups that are in conflict with the taxonomy are listed in Table 1.

NNv2 contains 91 species-groups at the fine level, compared to 56 in NNv1. Most fine level species-groups correspond to a single species, although some contain multiple closely related species, while in other cases multiple species-groups represent different clades of the same species (although this could be described or undescribed cryptic variation within a species or species complex). Table 1 lists all fine level species-groups that do not have one-to-one correspondence with a single species. Supplementary Table S1 contains individual level information for the samples in NNv2. Supplementary Figures S2–S7 show which pairwise distances meet the intermediate and fine level thresholds.

### Assignment of unrepresented species

The NNv2 reference index improves on the number of species that are represented compared to the NNv1 reference index, but there is some way to go to represent the complete genus *Anopheles* (500 species). Until we have most species represented in the reference index, it remains important to understand what happens if we try to assign an individual where its species is not represented yet in the reference index. Assuming that sufficiently many targets are amplified (≥10), the sample will be either assigned at the fine level, the intermediate level, the coarse level or not at any level. Among the species that are not represented in NNv1, but are represented in NNv2, we find examples of each of these scenarios.

#### Assignment to closely related species at fine level

**FIGURE 3:**
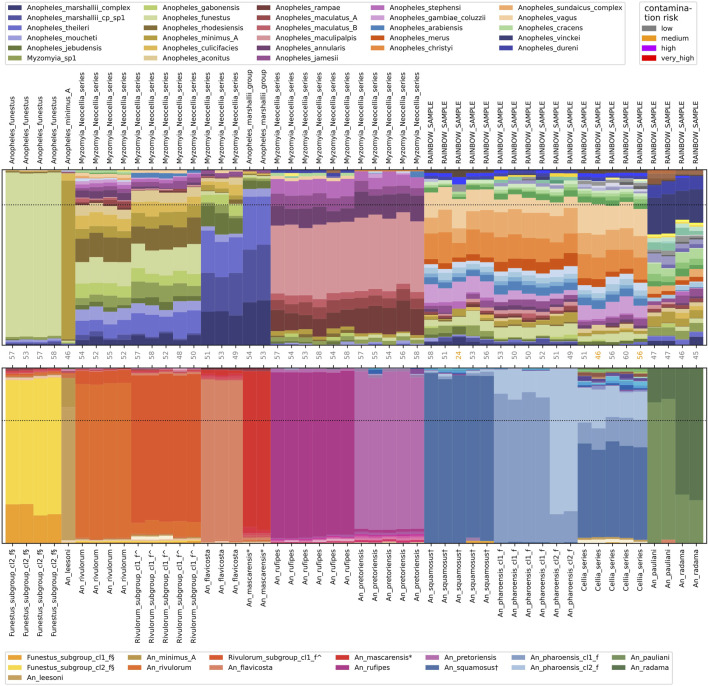
Resolved and persisting rainbow samples. Nearest neighbour assignment proportions using NNv1 (top) and using NNv2 (bottom) for 50 samples. Samples are labelled by their species call from the respective version of the reference index. The number of recovered targets and the contamination risk per sample is indicated along the horizontal axis between the two plots. The horizontal dotted lines indicate the assignment thresholds (0.8 for NNv1; 0.7 for NNv2).

Funestus_subgroup_cl2_f§ in NNv2 contains *An. longipalpis* and *An. parensis* and with NNv1 these samples were assigned to Anopheles_funestus at the fine level. Similarly, with NNv1 *An. leesoni* was assigned to Anopheles_minimus_A at the fine level (Figure 3). In NNv1, *An. funestus* and *An. minimus* are the only representatives of their respective subgroups, meaning that even though the samples we try to assign are of a different species, they are much closer to Anopheles_funestus and Anopheles_minimus_A respectively than to anything else in NNv1 and hence get assigned at the fine level to the incorrect species. NNv2 contains the fine level species-groups representing *An. longipalpis* and *An. parensis* (together represented as Funestus_subgroup_cl2_f§) and *An. leesoni* (An_leesoni). At the intermediate level, Funestus_subgroup_cl2_f§ belongs to the Funestus_subgroup and An_leesoni belongs to the Minimus_subgroup. Using NNv2, we correctly assign *An. longipalpis, An. parensis* and *An. leesoni* mosquitoes to their corresponding species-groups.

Two other species from the Funestus Subgroup, *An. vaneedeni* and *An. funestus-like*, were also assigned to Anopheles_funestus with NNv1. These species are more closely related to *An. funestus* than *An. longipalpis* and *An. parensis* are (Small et al., 2020) and in fact the normalised *k-*mer distance of *An. vaneedeni* and *An. funestus-like* to *An. funestus* is below the 0.1 fine level threshold. Therefore, these three species are now represented by a single fine level species-group: Funestus_subgroup_cl1_f§. So compared to NNv1, the new NNv2 reference index does not only add new fine level species-groups, but it also reevaluates the certainty of the labels and can change a label that represents a single species, suggesting confident assignment to that species, into a label that represents multiple species and no confident assignment to a single species. In the case of the Funestus Subgroup this uncertainty is somewhat alleviated by the likely restricted species ranges of all species other than *An. funestus* (Dia et al., 2013).

#### Assignment to group at intermediate level

In NNv1, *An. mascarensis,* a *s*pecies endemic from Madagascar and Comoros (Meillon, 1947), was most similar to Anopheles_marshallii_complex, Anopheles_marshallii_cp_sp1 and Anopheles_theileri and it was assigned at the intermediate level to the Anopheles_marshallii_group, which contains those three fine level species-groups (Figure 3). NNv2 contains an An_mascarensis* species-group and the samples now get assigned to it at the fine level. At the intermediate level, the An_mascarensis* species-group is part of the Marshallii_group, consistent with the NNv1 assignment results.

#### Assignment to series at coarse level

*An. pretoriensis* could only be assigned at the coarse level to the Myzomyia_Neocellia_series in NNv1. From the assignment proportions, we see that it is most closely related to Anopheles_maculipalpis, Anopheles_rampae and Anopheles_annularis, but it did not meet the assignment threshold at the fine or intermediate level (Figure 3). NNv2 contains an An_pretoriensis species-group and the samples get assigned to it at the fine level.

#### Rainbow samples

The assignment proportions for *An. squamosus* in NNv1 are distributed over many different species, resulting in a colourful pattern in the assignment proportions plot, which we refer to as ‘rainbows’. In NNv1 *An. squamosus* samples could not be assigned at any level. *An. squamosus* is in the Cellia Series in the subgenus *Cellia* and NNv1 did not contain any samples representing the Cellia Series, i.e., *An. squamosus* is quite distant from all the species in NNv1. NNv2 contains an An_squamosus† fine level species-group and the samples get assigned at the fine level.

Even if a sample does not get assigned at all, or only at the coarse level, the assignment proportions can still be used to group samples with similar rainbow patterns together, which can be helpful in order to find out what species they are. The NNv1 assignment proportions in Figure 3 show at least four different rainbow patterns: An_rivulorum and Rivulorum_subgroup_cl1_f^ (and maybe An_flavicosta because it has similar colours but quite different proportions); An_rufipes and An_pretoriensis; An_squamosus†, An_pharoensis_cl1_f and An_pharoensis_cl2_f; An_pauliani and An_radama. However, with the representation of these species in NNv2, we find that each of these four rainbow patterns in fact represents at least two different fine level species-groups.

A few samples can only be assigned at the coarse level even with NNv2. This can be due to contamination, in which case we would typically flag the samples as having an increased contamination risk. Moreover, if the species of both the sample and the contaminant are represented in the reference index, we would typically see high assignment proportions for these two species and very low assignment proportions for all other species. If we believe that the pattern we observe is not driven by contamination, and in particular if we see multiple samples with a repeated rainbow pattern, it is likely that these samples are of a species or population which is not represented in the reference index; that is what we suspect for the five Cellia_series samples in Figure 3 (see next section).

### Madagascar case study

**FIGURE 4:**
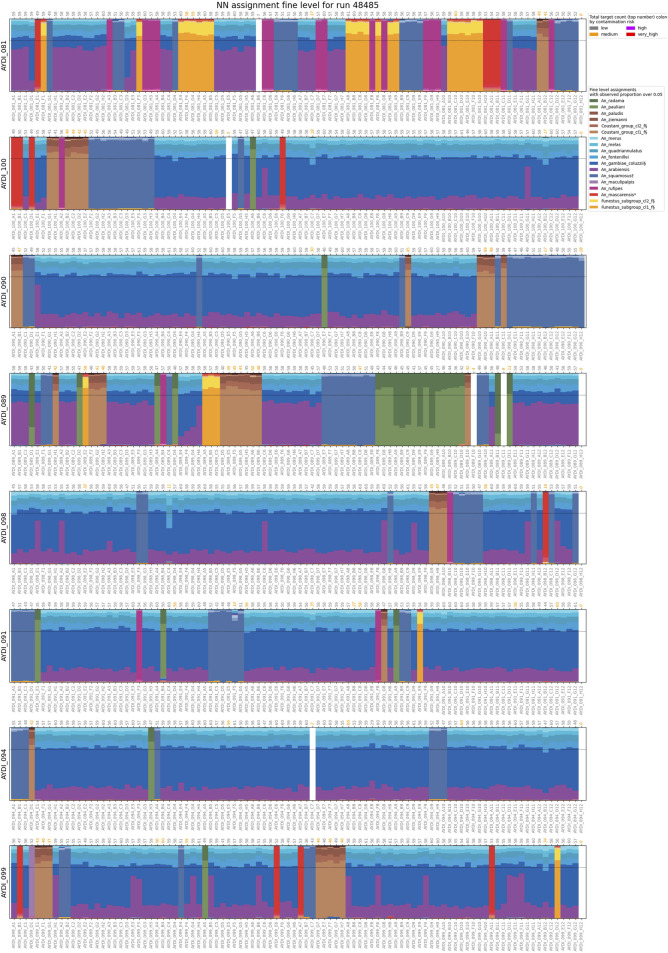
Assignment proportions for 760 mosquitoes from Madagascar. Assignment proportions for each individual are shown as vertical bars. Samples are grouped by sequencing plate (eight 96-well plates, the final well in each plate is a negative control). Sample names are shown at the bottom of each plate, the number of targets recovered at the top of each plate. The number of targets are coloured according to the contamination risk of the sample. The dotted horizontal line indicates the assignment threshold of 0.7.

**FIGURE 5:**
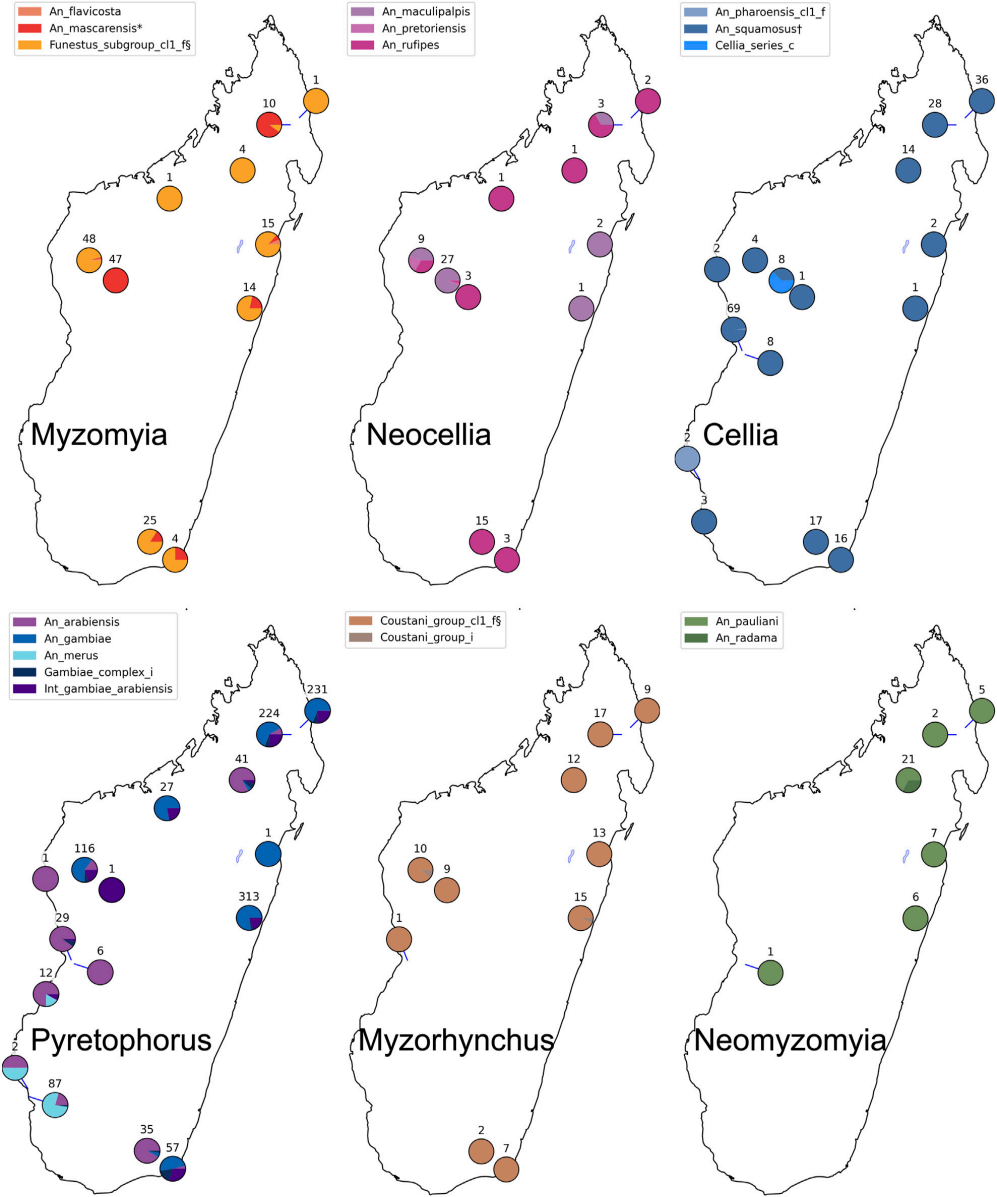
Species assignments for 1789 mosquitoes from Madagascar. The maps show the species of the mosquitoes collected at each location; each map presents a different coarse level species-group. The number displayed at each collection location refers to the number of individuals collected there for the focal coarse level species-group.

We performed species assignment for 1789 mosquitoes from different collection sites in Madagascar (Figures 4, 5; Supplementary Table S3). Assignment proportions for 760 individuals that were sequenced in one MiSeq run are displayed in Figure 4; this visual summary provides a quick overview of the species that are present. Of the entire dataset from Madagascar, 20 individuals (1.1%) had fewer than 10 targets amplified and hence were not run through NNoVAE. All other samples could be assigned at least at the coarse level. Five individuals were assigned to Cellia_series at the coarse level and could not be assigned at the intermediate level; we believe that these individuals represent a species within the Cellia Series that is not represented in NNv2. They were morphologically identified to be *An. coustani* (n = 2) and *An. squamosus* (n = 3). The majority of samples that could not be assigned at fine level are samples in the Gambiae Complex. We previously found that the NN method cannot confidently distinguish between very closely related species (e.g., *An. gambiae* and *An. coluzzii*) within species complexes and therefore we designed a follow-up method using a variational auto-encoder [VAE, see Boddé et al. (2022)] to identify species within the Gambiae Complex. Using this method, we identified 166 *An. arabiensis* and 660 *An. gambiae* specimens and 229 individuals (12.8%) were identified as intermediates between these two species. We believe that in most cases, these intermediates reflect the uncertainty of the VAE assignment method, rather than true hybrids between species. Although the NN assignment threshold is usually not reached for *An. arabiensis* and *An. gambiae*, the NN assignment proportions do show a different pattern for *An. arabiensis* (e.g., AYDI_098_E1 in Figure 4) and *An. gambiae* (e.g., AYDI_098_F1 in Figure 4). All other 1,477 mosquitoes (82.6%) could be assigned at the fine level. Figure 5 shows the collection locations of all individuals, split by coarse level series and coloured by fine level species.

### Population structure within the cellia series

In NNv2 the coarse level species-group Cellia_series consists of three fine level species-groups: An_squamosus† with samples from Ghana, Gabon, Madagascar and Tanzania; An_pharoensis_cl1_f with samples from Senegal, Ghana, Madagascar and Tanzania; and An_pharoensis_cl2_f with samples that occur sympatrically in Tanzania with An_pharoensis_cl1_f. Although there is some population structure visible within *An. squamosus*, we found that if we included two separate clusters, many samples would not meet the assignment threshold for either clade at the fine level and could only be assigned to the An_squamosus_i species-group at the intermediate level, which contains both clades. For *An. pharoensis,* almost all individuals were clearly assigned to one of the clades at the fine level. Moreover, the two *An. pharoensis* clades occur sympatrically in Tanzania, while the two *An. squamosus* clades occur on either side of the continent and hence the latter could be due to strong geographic population structure.

**FIGURE 6:**
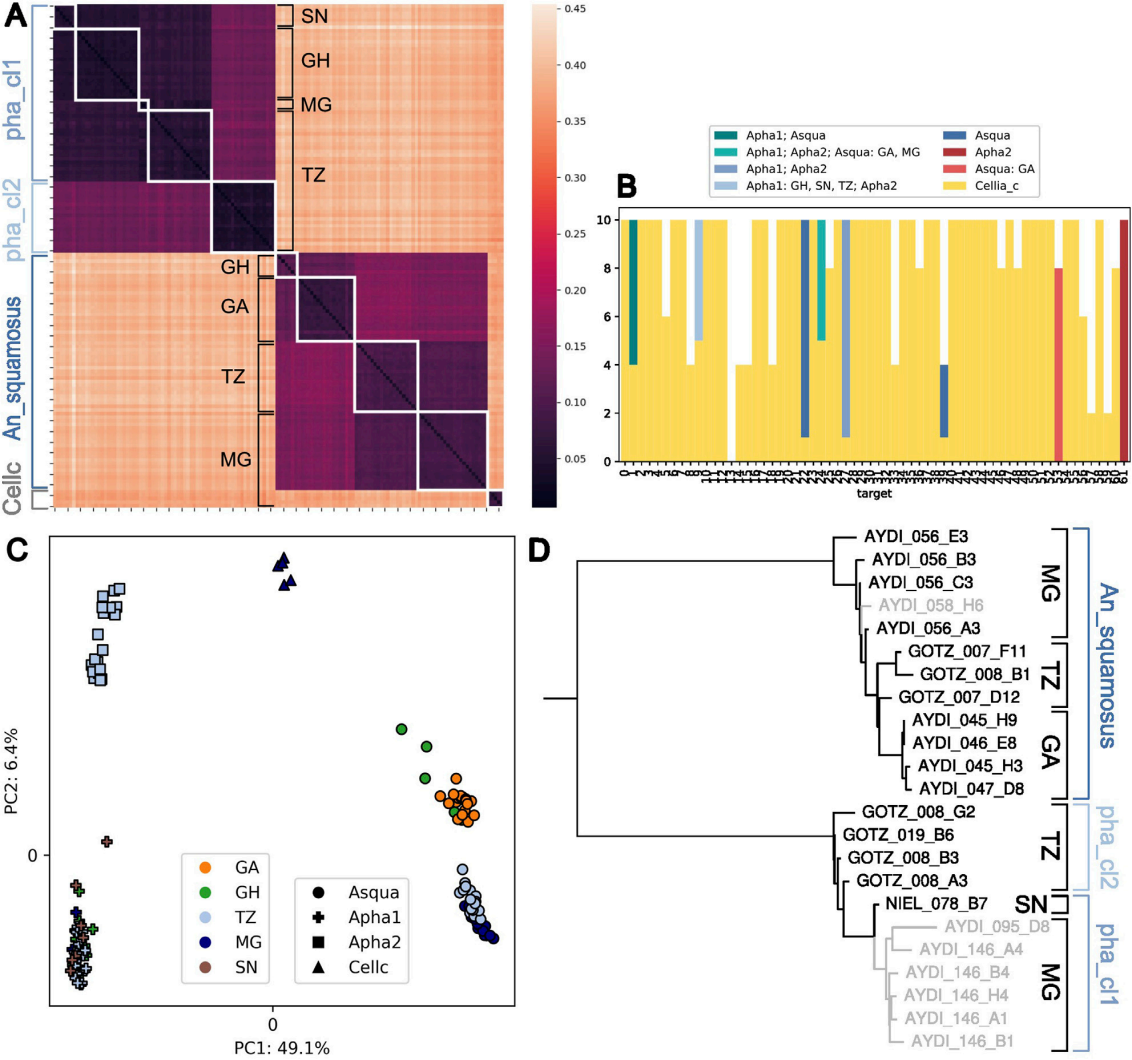
Structure and diversity within the Cellia Series. **(A)** Heatmap of pairwise distances between samples in the Cellia Series. Sample labels are a concatenation of their species assignment (Asqua: An_squamosus†, Apha1: An_pharoensis_cl1_f, Apha2: An_pharoensis_cl2_f at fine level and Cellc: Cellia_series at coarse level) and the country of collection (SN: Senegal, GH: Ghana, GA: Gabon, TZ: Tanzania, MG: Madagascar). White squares group together samples with the same label. **(B)** Haplotype sharing of Cellia_series individuals. We treat each distinct target sequence carried by the individuals that could not be assigned at the fine level as a haplotype and check for each haplotype in which other populations it occurs. The bar plot shows the number of alleles (2 per individual) with their sharing pattern. **(C)** Projection along the first two principal components computed on variable 8-mers summed over all targets. The percentage of explained variance is shown in the axis label. **(D)** ITS2 three of mosquitoes in the Cellia Series. Those in black have been assigned using NNv2 to the species-group indicated by the brackets on the right (pha_cl2: An_pharoensis_cl2_f, pha_cl1: An_pharoensis_cl1_f). For those in grey we do not have ANOSPP data yet. The grey sample in the *An. squamosus* clade was not identified morphologically; of the lower six grey samples, four were morphologically identified as *An. pharoensis* and two as *An. squamosus*. The collection country is indicated with black brackets.

Most samples in the Cellia Series can be assigned to a species-group at the fine level, except five samples from Madagascar that are assigned at the coarse level to the Cellia_series, but could not be assigned at the intermediate or fine level. Pairwise distances show that they are clearly distinct from the three fine level species-groups in the Cellia series (Figure 6A). If we treat each unique target sequence as a separate haplotype and record in which other populations in the Cellia Series these haplotypes occur, we find that these five fine level unassigned samples do not share exact sequence with any other samples in the Cellia series for the majority of the 62 targets (Figure 6B). They also form their own cluster in the PCA which is clearly different from An_squamosus†, An_pharoensis_cl1_f, and An_pharoensis_cl2_f (Figure 6C).

Pairwise distances reveal considerable geographic structure between the An_squamosus† samples; we see one cluster containing individuals from Ghana and Gabon and another cluster with individuals from Tanzania and Madagascar (Figures 6A, C). This shows that the ANOSPP sequence captures a geographic signal within *An. squamosus*.

Sanger sequencing using only the ITS2A forward primer yielded incomplete sequences for *An. squamosus* and *An. pharoensis* and resulted in sequence length variation between individuals. The consensus sequences from combining the chromatograms sequenced from both the forward and reverse primer resulted in more consistent sequence length between individuals. The ITS2 tree from Cellia individuals shows a clear split between *An. squamosus* and *An. pharoensis* individuals (Figure 6D). Unfortunately, we do not have any ITS2 information for the five Cellia_series individuals that could only be assigned at coarse level. We have ITS2 sequences for An_pharoensis_cl2_f individuals from Tanzania, but not for any sympatric An_pharoensis_cl1_f individuals; in fact the only confirmed An_pharoensis_cl1_f individual with ITS2 information is from Senegal and it forms a clade together with six individuals from Madagascar for which we have not yet generated ANOSPP data. However, we have only encountered An_pharoensis_cl2_f in Tanzania and we have previously sequenced three An_pharoensis_cl1_f mosquitoes from Madagascar, so it is likely that these individuals in the tree will be assigned to An_pharoensis_cl1_f. In summary, ITS2 barcodes clearly differentiate between *An. pharoensis* and *An. squamosus* and there is a hint of the geographic structure in *An. squamosus* and the two different clades in *An. pharoensis*, but the structure does not stand out as clearly using ITS2 data as it does using ANOSPP, supporting ANOSPP as a valuable approach to understand within species-group structure.

## Discussion

Historically, the classification of the species of the genus *Anopheles* is based on morphological characteristics such as wing spots, leg coloration and head anatomy. DNA based phylogenetic studies report cases of concordance as well as discordance (Harbach, 2013; Sallum et al., 2020) with the morphological taxonomy. We find that the coarse structure of the pairwise distances between samples in NNv2 mostly agrees with the series level (subgenus level for *Kerteszia* and *Nyssorhynchus*) of the *Anopheles* taxonomy. However, we find that the Cellia and Pyretophorus Series cannot be separated; it would be interesting to investigate the phylogeny of the species in those series using complete genome assemblies which are underway. The Neomyzomyia Series does not form a single cluster, consistent with reports of it being non-monophyletic (Foley et al., 1998; Harbach, 2013; Makunin et al., 2022). Moreover, three species which, according to the taxonomy, belong to the Neomyzomyia Series, *An. rhodesiensis*, *An. mascarensis* and *An. jebudensis*, cluster with the Myzomyia Series in our results.

Series are usually separated based on the cibarial armature, a structure located near the mouth (Gillies and De Meillon, 1968). It has previously been questioned whether the cibarial armature is a reliable characteristic to differentiate between taxonomic series in the genus *Anopheles* (Rahola et al., 2014). In light of our results, it would be interesting to investigate the evolutionary origin of the distinctive features or the cibarial armature and other morphological characteristics used for taxonomic classification with whole genome data.

One of the powers of NNoVAE is that when a species is not represented in the reference index, it gives an indication which species in the reference index are closest to it. Comparing the assignment results generated with different reference index versions, we observed the different scenarios that can occur when a species is not represented in the reference index. If it is also not represented by any close relatives in the reference index, it will only be assigned at the coarse level or it will not be assigned at all. In these cases, the assignment proportions form a characteristic rainbow pattern, which acts like a fingerprint; it is possible to group samples according to the rainbow patterns, even if it is not yet possible to put species names on these groups. We found that samples with similar rainbow patterns are indeed of related species, but it is difficult to distinguish the rainbow patterns of closely related species if neither of them are present in the reference index.

One of the remaining rainbow patterns we see in assignments with NNv2 are samples assigned at the coarse level to the Cellia Series. These samples likely represent another species within the Cellia Series; candidates are *An. argenteolobatus*, which has a wide geographic spread, from Burkina Faso to Tanzania to South-Africa (Irish et al., 2020), but has not previously been recorded in Madagascar, or *An. cydippis*, which has previously been found on Madagascar (Andrianaivolambo et al., 2010), although we might expect this species to be more similar to *An. squamosus* than what we see for these samples. In our data we see considerable population structure within the other species in the Cellia Series. In Tanzania, *An. pharoensis* clearly forms two separate clusters. There are several sites where these two clades occur sympatrically. It is possible that the Tanzania-specific clade represents cryptic variation within *An. pharoensis* or that it is in fact another species in the Cellia Series, e.g., *An. swahilicus*, which occurs in Tanzania and Kenya (Irish et al., 2020), *An. argeneolobatus* or *An. cydippis*, although the latter species we would rather expect to be closer to *An. squamosus* than *An. pharoensis* based on morphological taxonomy (Coetzee, 2020). *An. squamosus* displays strong geographic structure. This result shows that ANOSPP can be a useful tool to identify which species display strong population structure, which can be used to design follow up studies for species of interest. One has to bear in mind that all ANOSPP targets together amount to approximately 10 kb of sequence, so if population structure is driven by only a fraction of the genome it is possible that none of the ANOSPP targets are close enough to tag it. Nevertheless, here we demonstrate that ANOSPP captures geographic structure within *An. squamosus* and previously it has been shown that it captures geographic structure within *An. gambiae, An. coluzzii* and *An. arabiensis* (Boddé et al., 2022).

Here we presented an updated reference index for NNoVAE, NNv2, together with a clarified approach for future updates as we generate data for species that are not yet represented in NNv2. The updated reference index makes it possible to assign many more species with the NN method. Because the assignment threshold has been decreased compared to the earlier NNoVAE version, even some species within the Gambiae Complex, e.g., *An. melas* and *An. merus*, can already be assigned with the NN method without the need to be run through the Gambiae complex VAE. On the other hand, we found that some species-groups that we thought represented a single species actually represent several species that cannot be distinguished by the NN method, e.g., Funestus_subgroup_cl1_f§ represents three species in the Funestus Subgroup: *An. funestus, An. funestus-like* and *An. vaneedeni*. We plan to implement a tailored follow up method for each group of species that the NN method cannot differentiate. For groups where we have many samples available of known species identity, we could design a VAE like we did for the Gambiae Complex (Boddé et al., 2022). For groups with fewer available samples it would be more feasible to look, for example, for diagnostic *k-*mers for each of the known species, similar in spirit to the Ancestry Informative Markers (Anopheles gambiae 1000 Genomes Consortium et al., 2017).

## Data Availability

The original contributions presented in the study are publicly available. This data can be found on the European Nucleotide Archive (ENA), per-sample accessions are available in Supplementary Tables S1–S4.

## Supplementary material

SUPPLEMENTARY FIGURE S1 Pairwise distances between samples in NNv2. The heatmap shows whether the pairwise *k*-mer distances between all individuals in NNv2 are below the threshold values for the coarse (<0.5, shown in eggplant), intermediate (<0.3, shown in mauve rose) and fine (<0.1, shown in light peach) assignment levels. Individuals are ordered the same as in Figure 1 and are labelled by the fine-level species groups (labels are shown only for every third sample because of size restrictions). The orange and yellow and green squares indicate the fine, intermediate and coarse level species groups respectively.

SUPPLEMENTARY FIGURE S2 Pairwise distances between samples in the Myzomyia Series. The heatmap shows whether the pairwise *k*-mer distances between all Myzomyia Series individuals in NNv2 are below the threshold values for the coarse (<0.5, shown in eggplant), intermediate (<0.3, shown in mauve rose) and fine (<0.1, shown in light peach) assignment levels. Individuals are ordered the same as in Figure 1 and are labelled by the fine-level species groups (labels are shown only for every second sample because of size restrictions). The orange and yellow squares indicate the fine and intermediate level species groups respectively.

SUPPLEMENTARY FIGURE S3 Pairwise distances between samples in the Neocellia Series. The heatmap shows whether the pairwise *k*-mer distances between all Neocellia Series individuals in NNv2 are below the threshold values for the coarse (<0.5, shown in eggplant), intermediate (<0.3, shown in mauve rose) and fine (<0.1, shown in light peach) assignment levels. Individuals are ordered the same as in Figure 1 and are labelled by the fine-level species groups (labels are shown only for every second sample because of size restrictions). The orange and yellow squares indicate the fine and intermediate level species groups respectively.

SUPPLEMENTARY FIGURE S4 Pairwise distances between samples in the Cellia, Pyretophorus and Paramyzomyia Series. The heatmap shows whether the pairwise *k*-mer distances between all individuals from the Cellia, Pyretophorus and Paramyzomyia Series in NNv2 are below the threshold values for the coarse (<0.5, shown in eggplant), intermediate (<0.3, shown in mauve rose) and fine (<0.1, shown in light peach) assignment levels. Individuals are ordered the same as in Figure 1 and are labelled by the fine-level species groups (labels are shown only for every second sample because of size restrictions). The orange, yellow and green squares indicate the fine, intermediate and coarse level species groups respectively.

SUPPLEMENTARY FIGURE S5 Pairwise distances between samples in the Neomyzomyia Series. The heatmap shows whether the pairwise *k*-mer distances between all Neomyzomyia Series individuals in NNv2 are below the threshold values for the coarse (<0.5, shown in eggplant), intermediate (<0.3, shown in mauve rose) and fine (<0.1, shown in light peach) assignment levels. Individuals are ordered the same as in Figure 1 and are labelled by the fine-level species groups (labels are shown only for every second sample because of size restrictions). The orange and yellow squares indicate the fine and intermediate level species groups respectively.

SUPPLEMENTARY FIGURE S6 Pairwise distances between samples in the Christya, Anopheles and Myzorhynchus Series. The heatmap shows whether the pairwise *k*-mer distances between all individuals from the Christya, Anopheles and Myzorhynchus series in NNv2 are below the threshold values for the coarse (<0.5, shown in eggplant), intermediate (<0.3, shown in mauve rose) and fine (<0.1, shown in light peach) assignment levels. Individuals are ordered the same as in Figure 1 and are labelled by the fine-level species groups (labels are shown only for every second sample because of size restrictions). The orange, yellow and green squares indicate the fine, intermediate and coarse level species groups respectively.

SUPPLEMENTARY FIGURE S7 Pairwise distances between samples in the subgenera *Kerteszia* and *Nyssorhynchus*. The heatmap shows whether the pairwise *k*-mer distances between all individuals from the subgenera *Kerteszia* and *Nyssorhynchus* in NNv2 are below the threshold values for the coarse (<0.5, shown in eggplant), intermediate (<0.3, shown in mauve rose) and fine (<0.1, shown in light peach) assignment levels. Individuals are ordered the same as in Figure 1 and are labelled by the fine-level species groups. The orange, yellow and green squares indicate the fine, intermediate and coarse level species groups respectively.
