# Supplementary figures and images for: Improved species assignments across the entire Anopheles genus using targeted sequencing
Source: Front Genet. 2024 Sep 19;15:1456644. doi: 10.3389/fgene.2024.1456644 (PMC11446804; doi:10.3389/fgene.2024.1456644)

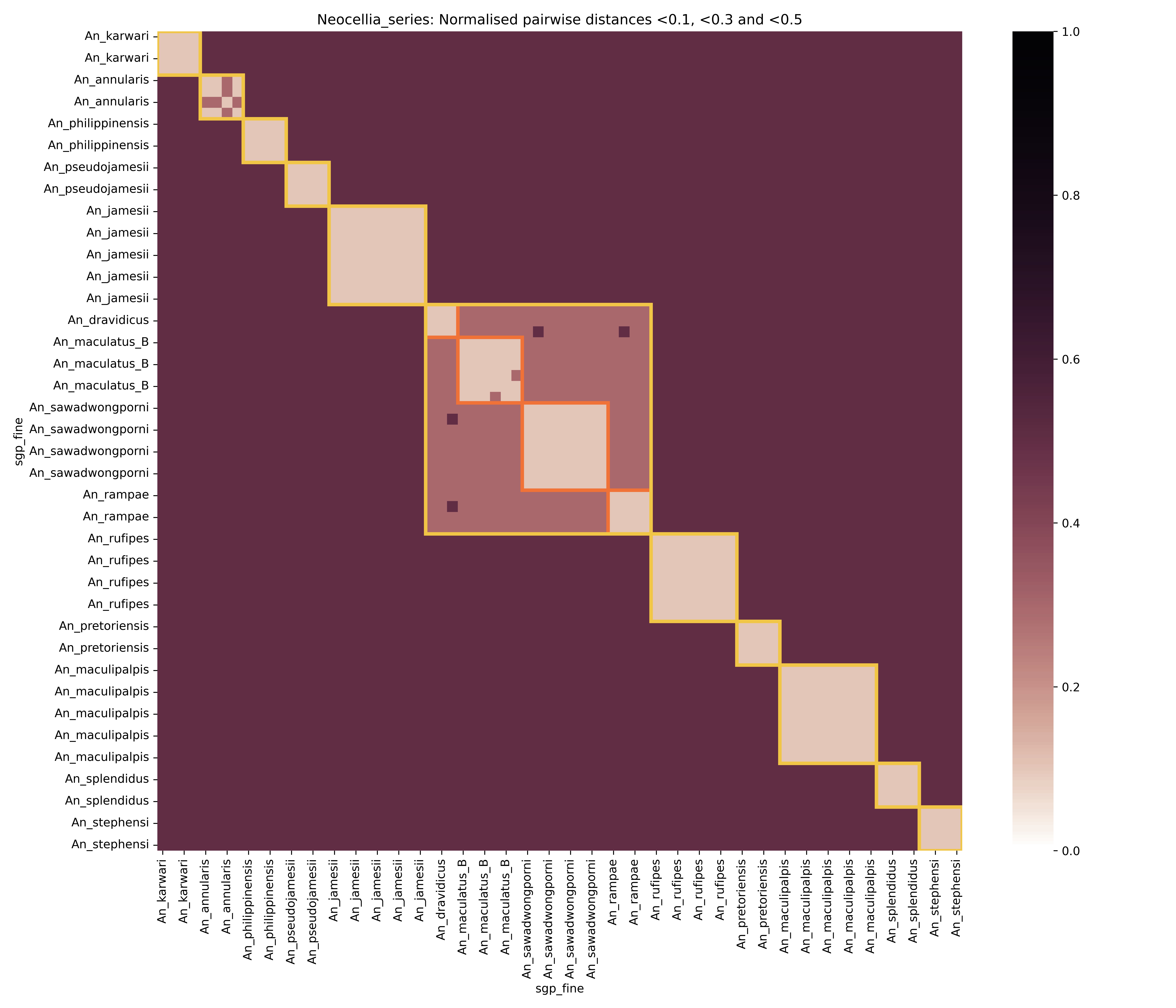

Supplement: Supplementary file 1 [file Image3.JPEG]

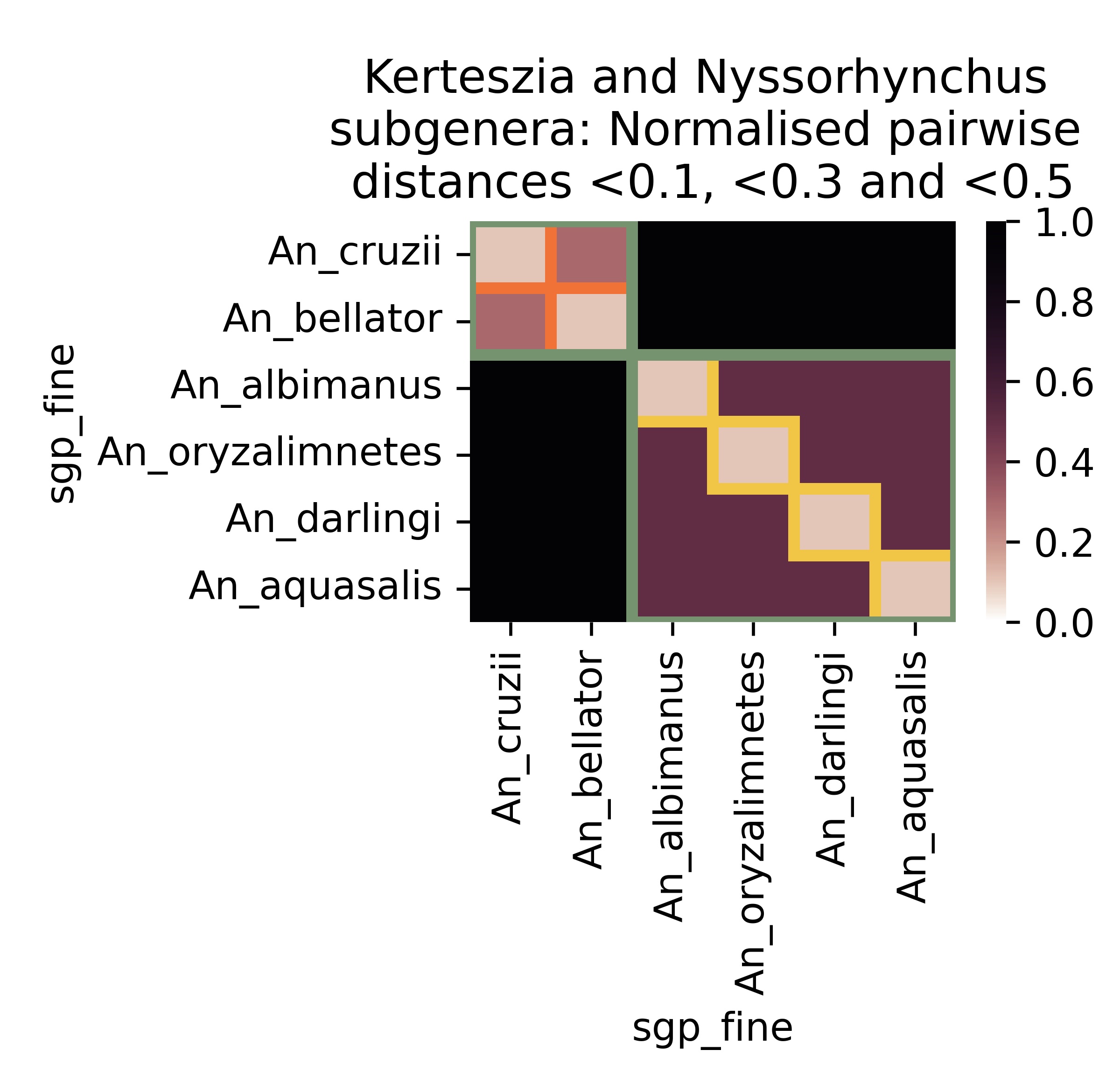

Supplement: Supplementary file 6 [file Image7.JPEG]

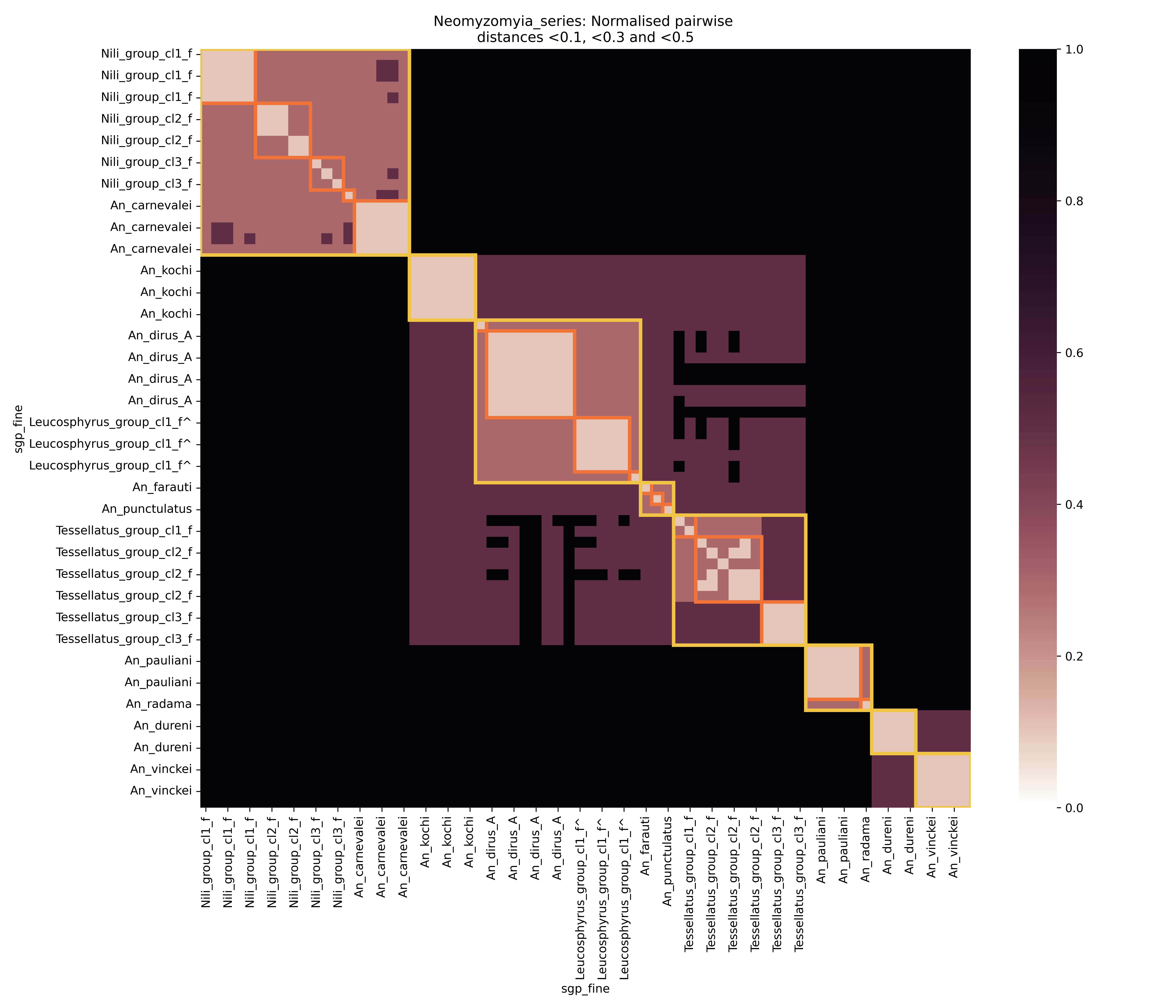

Supplement: Supplementary file 8 [file Image5.JPEG]

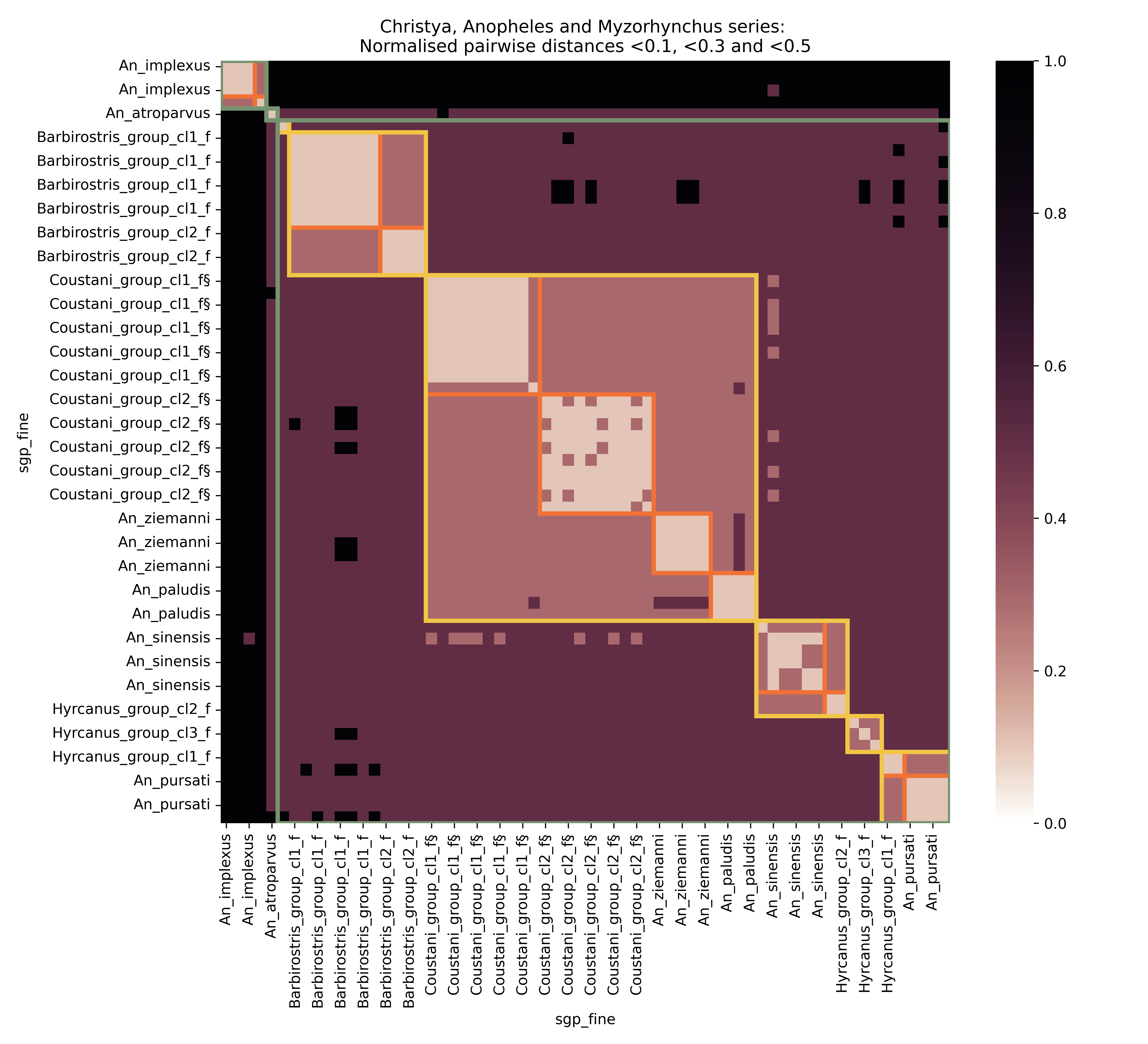

Supplement: Supplementary file 11 [file Image6.JPEG]
